# Laser‐Polarization‐Induced Anisotropy Enhances Protein Crystallization

**DOI:** 10.1002/anie.202501827

**Published:** 2025-03-17

**Authors:** Tien Chen, Hirotsugu Hiramatsu, Shuichi Toyouchi, Teruki Sugiyama

**Affiliations:** ^1^ Department of Applied Chemistry and Center for Emergent Functional Matter Science National Yang Ming Chiao Tung University Hsinchu 300093 Taiwan; ^2^ Research Institute for Light‐induced Acceleration System (RILACS) Osaka Metropolitan University Sakai 599–8570 Japan; ^3^ Division of Materials Science Graduate School of Science and Technology Institution Nara Institute of Science and Technology Ikoma 630‐0192 Japan

**Keywords:** Anisotropy, Fluorescence anisotropy, Laser polarization, Optical trapping, Protein crystallization

## Abstract

Protein crystallization plays a critical role in structural biology and drug development, driving extensive research into its control. We present a novel approach for manipulating protein crystallization by controlling the anisotropy of the high‐concentration domain (HCD) composed of hen egg‐white lysozyme (HEWL) through laser polarization in optical trapping. The anisotropy of the HCD was assessed through fluorescence anisotropy measurements and polarized Raman spectroscopy. The results revealed that linear polarization significantly enhances crystallization efficiency by inducing anisotropy of the HCD and promoting nucleation. In contrast, circular polarization resulted in weak anisotropy with minimal crystallization efficiency. This study highlights the crucial role of protein molecule anisotropy in crystallization under optical trapping conditions, providing a new strategy to optimize laser conditions for various protein molecules and pave the way for the development of innovative crystallization techniques, potentially revolutionizing structural biology and drug discovery efforts.

## Graphical abstract

The polarization of the optical trapping laser has a significant impact on the crystallization of hen egg‐white lysozyme. The anisotropy of the high‐concentration domain composed of HEWL molecules/clusters plays a crucial role in the crystallization process. Linear polarization promotes crystallization by inducing strong anisotropy, whereas circular polarization leads to weak anisotropy and minimal enhancement of crystallization.

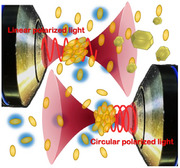

## Introduction

Protein crystallization, despite its pivotal role in structural biology and drug development, remains a challenge owing to the difficulty in controlling nucleation and crystal growth.^[^ ^1^, ^2^, ^3^, ^4^ ^]^ This challenge has driven extensive research into new methods for enhancing crystallization efficiency and understanding the underlying mechanisms. In particular, the role of molecular orientation and structural fluctuations in the nucleation process has garnered increasing attention. Building on the two‐step nucleation mechanism proposed by Vekilov et al.,^[^ ^5^, ^6^, ^7^ ^]^ which highlights the importance of density and structural fluctuations, this study investigates the impact of laser polarization on the anisotropy of protein molecules within a high‐concentration domain (HCD) generated by optical trapping.

Optical trapping is a promising method for protein crystallization that uses a tightly focused laser beam to manipulate small particles with high precision.^[^ ^8^, ^9^, ^10^, ^11^, ^12^ ^]^ The principle of optical trapping lies in the momentum transfer between photons and trapped particles Supporting Information (SI 1). We previously enhanced the crystallization efficiency of hen egg‐white lysozyme (HEWL) using optical trapping. By fine‐tuning the laser conditions, such as intensity and polarization, we controlled the rate of nucleation and crystal growth.^[^ ^13^, ^14^, ^15^ ^]^ In our recent study, we demonstrated that 100% crystallization of HEWL could be achieved within 1 h by optimizing the concentration of fluorescent‐labeled HEWL molecules at the focal point using optical trapping in linear polarization (LP) mode.^[^ ^16^ ^]^ Notably, HEWL crystallization is suppressed during laser irradiation but is triggered within 30 min after stopping the irradiation. These studies demonstrate that optical trapping can significantly influence the nucleation and growth rates of protein crystals, resulting in a much‐shortened crystallization time.

**Figure 1:**
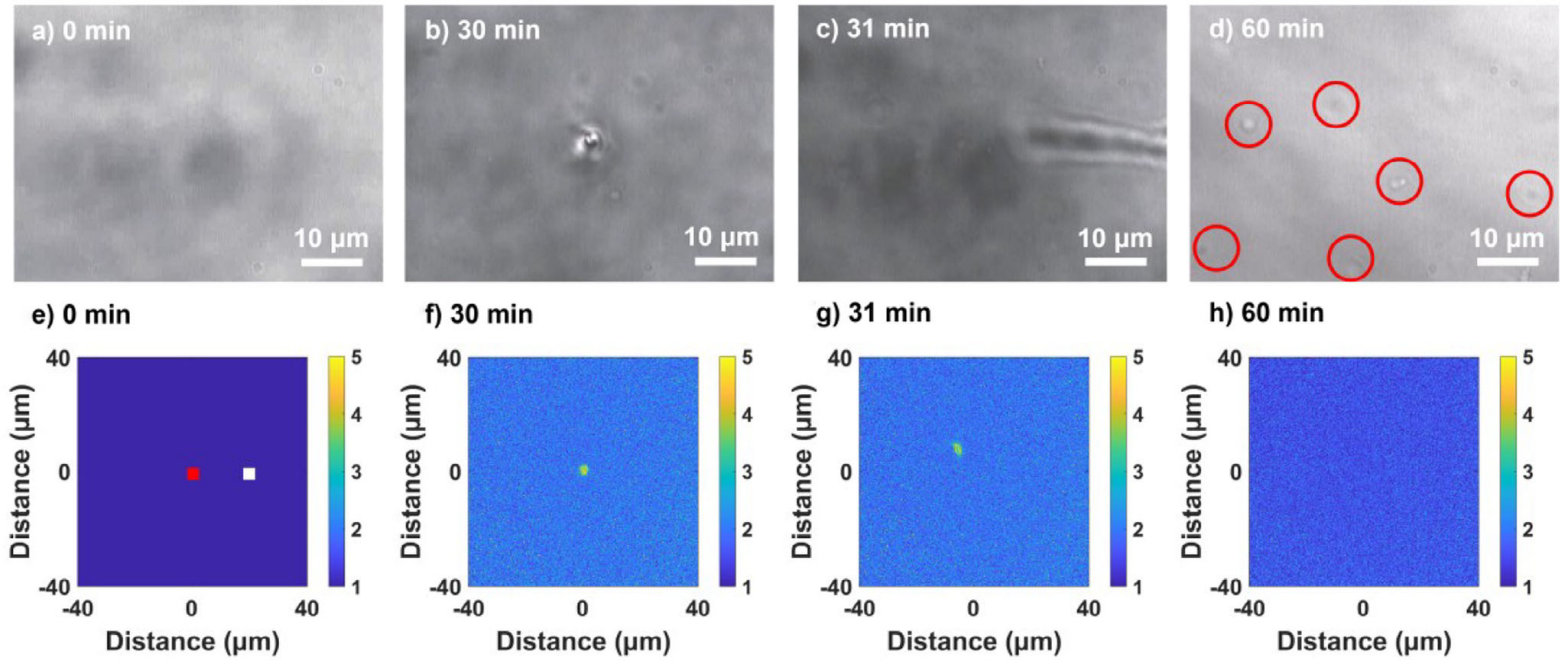
Transmission a–d) and fluorescence e–h) images showing the dynamics of trapping and condensation at (a) (e) 0 min, and (b) (f) 30 min during laser irradiation, and at (c) (g) 31 min (1 min after stopping laser irradiation), and (d) (h) 60 min after the cessation of laser irradiation. The red circles in (d) highlight the small HEWL condensates. The red and white dots in (e) represent the focal position and 20 µm away from the focal position, respectively.

Additionally, optical trapping generates the HCD at/around the focal spot, where the diffusion of HEWL molecules is significantly slowed, even after turning off the laser irradiation (Figure 1). This slow diffusion rate promotes crystallization by increasing the probability of protein–protein interactions and facilitating nucleation.^[^ ^16^ ^]^ Our findings have paved the way for the exploration of optical trapping as a versatile tool in protein crystallization research. In this work, we focus on the detailed dynamics and mechanism of crystallization behavior under optical trapping conditions, particularly the roles of anisotropy, cluster formation, and laser‐induced effects.

This study builds on our prior work by investigating the effects of the polarization modes of trapping lasers on the anisotropy and crystallization behavior of HEWL. By utilizing optical trapping, we aim to understand the mechanisms driving protein crystallization and explore the potential of this method for enhancing crystallization efficiency. This research will lead to the development of techniques applicable to investigate the crystallization dynamics and mechanism of other protein molecules.

## Results and Discussion

### HEWL Trapping and Condensation Dynamics: Transmission and Fluorescence Images during and after CP Laser Irradiation

To investigate the aggregation dynamics of HEWL under circularly polarized (CP) laser irradiation, a 1064 nm continuous‐wave laser at 1.0 W was employed as the trapping source. The laser was focused into a thin film (110–130 µm thickness) of HEWL solution (40 mg mL^−1^ HEWL, 2.7% (w/v) NaCl) at a point 20 µm above the bottom cover glass. To further analyze the concentration dynamics of HEWL molecules during optical trapping, a small amount of fluorescent dye‐labeled HEWL (F‐HEWL) was added to the HEWL sample solution (40 mg mL^−1^ HEWL, 2.7% (w/v) NaCl, 0.01 mg mL^−1^ F‐HEWL). The laser was irradiated for 30 min, followed by a 30 min observation period without irradiation. Figure 1 depicts the resulting temporal changes in transmission and fluorescence images, illustrating the trapping and condensation dynamics of HEWL during and after optical trapping.

Initially, the sample solution appeared homogeneous, with no discernible substance (Figure 1a). Upon laser irradiation, a condensate gradually formed at the laser focus, maintaining a consistent size of ≈3 µm (Figure 1b). While the condensate size remained stable, its transmittance decreased, indicating an increase in HEWL concentration within the condensate. Concurrently, fluorescence intensity increased not only at the focal point but also in the surrounding region (Figure 1f), signifying the formation of the HCD during the 30 min of CP irradiation. This increase in fluorescence intensity, which corresponds to an increase in HEWL concentration, will be quantified below.

Immediately after the laser was switched off, the condensate migrated away from the focal point (Figure 1c). Subsequently, small HEWL condensates emerged within 30 min of the post‐irradiation period (Figure 1d). These condensates gradually diminished and disappeared completely within 60 min after irradiation, returning the solution to its initial homogeneous state. Fluorescence images (Figure 1g,h) revealed a slow diffusion of the HCD during the post‐irradiation period. These observed behaviors under CP irradiation were phenomenologically consistent with those previously reported under LP irradiation.^[^ ^16^ ^]^

### Temporal Changes in Hen Egg‐White Lysozyme Concentration and Dependence of Hen Egg‐White Lysozyme Crystallization Probability under Different Laser Polarization Modes

To investigate the temporal changes in HEWL concentration at the focal point, we employed two laser polarization modes: LP and CP. A sample solution containing HEWL (40 mg mL^−1^), NaCl (2.7% w/v), and fluorescent dye‐labeled HEWL (F‐HEWL, 0.01 mg mL^−1^) was utilized. The concentration of HEWL was estimated using a calibration curve, which was created by measuring the fluorescence intensity of F‐HEWL/HEWL mixtures at various concentrations (Table S1). As shown in Figure S4, the calibration curve exhibited a linear relationship between the relative fluorescence intensity (1.0–8.5) and HEWL concentration (40–360 mg mL^−1^).

**Figure 2:**
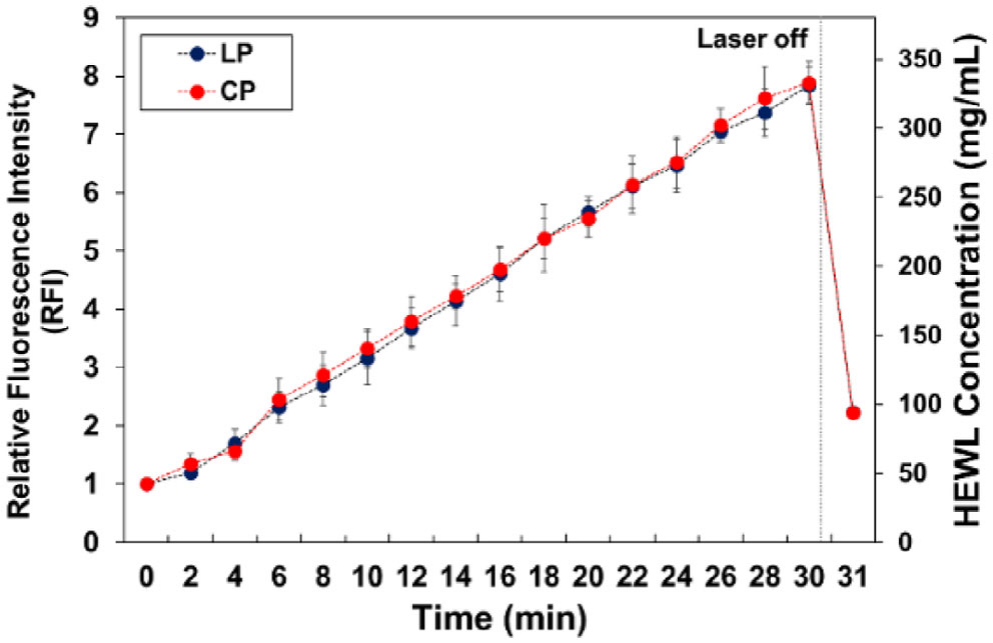
Temporal changes in the relative fluorescence intensity (RFI) and hen egg‐white lysozyme (HEWL) concentration at the focal spot during and after laser trapping. Black and red dots represent the temporal changes in the RFIs under linear polarization (LP) and circular polarization (CP) trapping laser irradiations, respectively. Error bars represent standard deviation from three independent experiments.

Figure 2 illustrates the temporal changes in relative fluorescence intensity (RFI) and corresponding HEWL concentration at the focal point during 30 min of laser irradiation in both LP and CP modes. The RFI values were calculated relative to the initial fluorescence intensity before laser irradiation. Additionally, the RFI measured 1 min after stopping the laser irradiation (31 min) is also shown. These measurements provide insights into the dynamics of HEWL concentration at the focal point under different laser polarization conditions. Figure 2 also shows that the HEWL concentration at the focal point increased almost linearly with laser irradiation time, regardless of the laser polarization modes, reaching ≈330 mg mL^−1^ (about eight times the pre‐irradiation concentration) after 30 min. This result indicates that the trapping efficiency of the HEWL molecules/clusters in the sample solution is almost independent of the trapping laser polarization modes.

For crystallization experiments, when the RFI at the focal point reached 6, 7, and 8, the laser irradiation was stopped, and the entire solution was examined for HEWL crystal generation using a low‐magnification objective lens (×10) for 30 min. Importantly, HEWL crystallization was never observed during laser trapping. Furthermore, spontaneous HEWL crystals typically form after at least two days under non‐irradiation conditions.

To understand the suppression of crystallization during laser irradiation, it is crucial to consider the potential temperature increase at the focal point due to laser absorption. As we previously reported,^[^ ^16^ ^]^ the estimated temperature increase under these conditions is ≈2 K. To further investigate the impact of temperature on HEWL crystallization, we conducted additional experiments at elevated temperatures.^[^ ^13^ ^]^ These experiments revealed that HEWL crystallization was not significantly affected by the temperature increase, indicating that it is not the primary cause for the observed suppression of crystallization.

**Figure 3:**
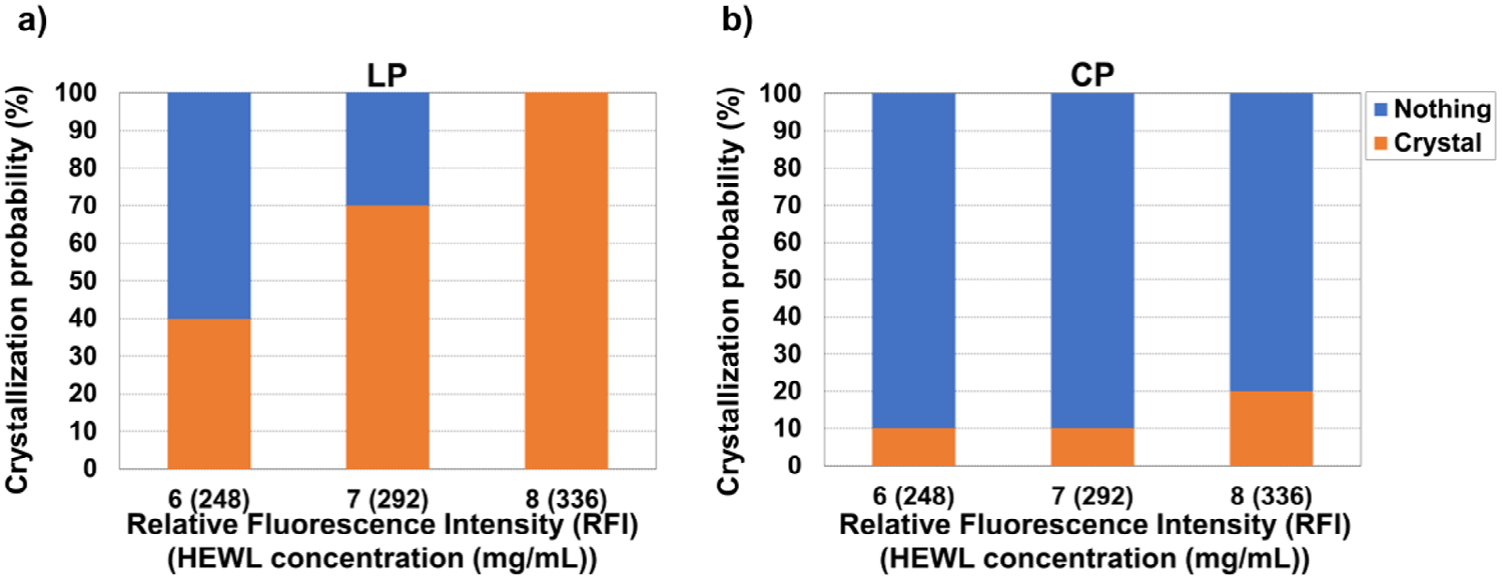
HEWL crystallization probability as a function of the RFI achieved at the focal spot under a) LP irradiation and b) CP irradiation. “Nothing” and “Crystal” in the legend indicate cases where crystallization did not be observed (blue bar) and observed (orange bar) under optical trapping conditions, respectively.

The polarization dependence of the HEWL crystallization probability was investigated by repeating 10 times for each polarization mode. The results are summarized in Figure 3. Under LP trapping laser irradiation conditions (Figure 3a), when the laser irradiation was stopped at RFI 6 (corresponding to HEWL concentration of 248 mg mL^−1^), the HEWL crystallization probability in 30 min post‐irradiation was 40%. Interestingly, the generated crystals were concentrated near the points projected vertically onto the cover glass from the focal point. As the RFI at the focal point increased, the HEWL crystallization probability increased, reaching 100% when the RFI reached 8 (corresponding to a HEWL concentration of 336 mg mL^−1^). In contrast, under CP trapping laser irradiation conditions, the HEWL crystallization probability at RFI 6 was only 10%. Even as the RFI increased, the HEWL crystallization probability barely changed, reaching only ≈20% even at RFI 8. Our experiments clearly demonstrated a significantly higher HEWL crystallization probability with LP trapping laser compared to CP trapping laser. These results strongly suggest that the main factor inducing the HEWL crystallization is not just the increase in concentration but also the molecular alignment induced by the polarization mode of the trapping laser.

### Fluorescence Anisotropy Analysis of Hen Egg‐White Lysozyme Solutions under Optical Trapping Conditions

To investigate the anisotropy of the condensate and the HCD, we measured the temporal changes in FA at the focal point and 20 µm away from the focal position. These points are indicated by the red and white dots in Figure 1e, respectively. FA is defined as the ratio of the fluorescence intensities measured when the polarization of the excitation laser is perpendicular (RFI_90_) and parallel (RFI_0_) to the polarization of the trapping laser:

(1) $$\mathrm{FA}=\frac{\mathrm{Relative}\;\mathrm{Fluorescence}\;\mathrm{Intensity}\;{\left(\mathrm{RFI}\right)}_{90}}{\mathrm{Relative}\;\mathrm{Fluorescence}\;\mathrm{Intensity}\;{\left(\mathrm{RFI}\right)}_{0}},$$

A schematic illustration of the experimental setup for FA measurements is shown in Figure S2. The excitation wavelength was 405 nm, and FA was evaluated from the fluorescence intensity at 453 nm. In the case of the CP trapping laser, RFI_90_ and RFI_0_ were defined as the horizontal and vertical directions of the excitation laser polarization, respectively (Figure S5). The FA values were corrected to 1 for the initial solution without trapping laser irradiation by assuming that the HEWL molecules are intrinsically isotropic (see Table S2). An FA value of 1 indicates that the condensate and the HCD are completely isotropic. Deviations from 1 indicate increasing anisotropy, with larger deviations corresponding to higher anisotropy. We confirmed that a solution adjusted to RFI = 8 (corresponding to 330 mg mL^−1^) without optical trapping exhibited an FA value of 1, indicating that the increase of the HEWL concentration did not induce any anisotropy. The temporal changes in FA were measured by monitoring RFI_90_ and RFI_0_ every 10 min during LP and CP trapping laser irradiation.

**Figure 4:**
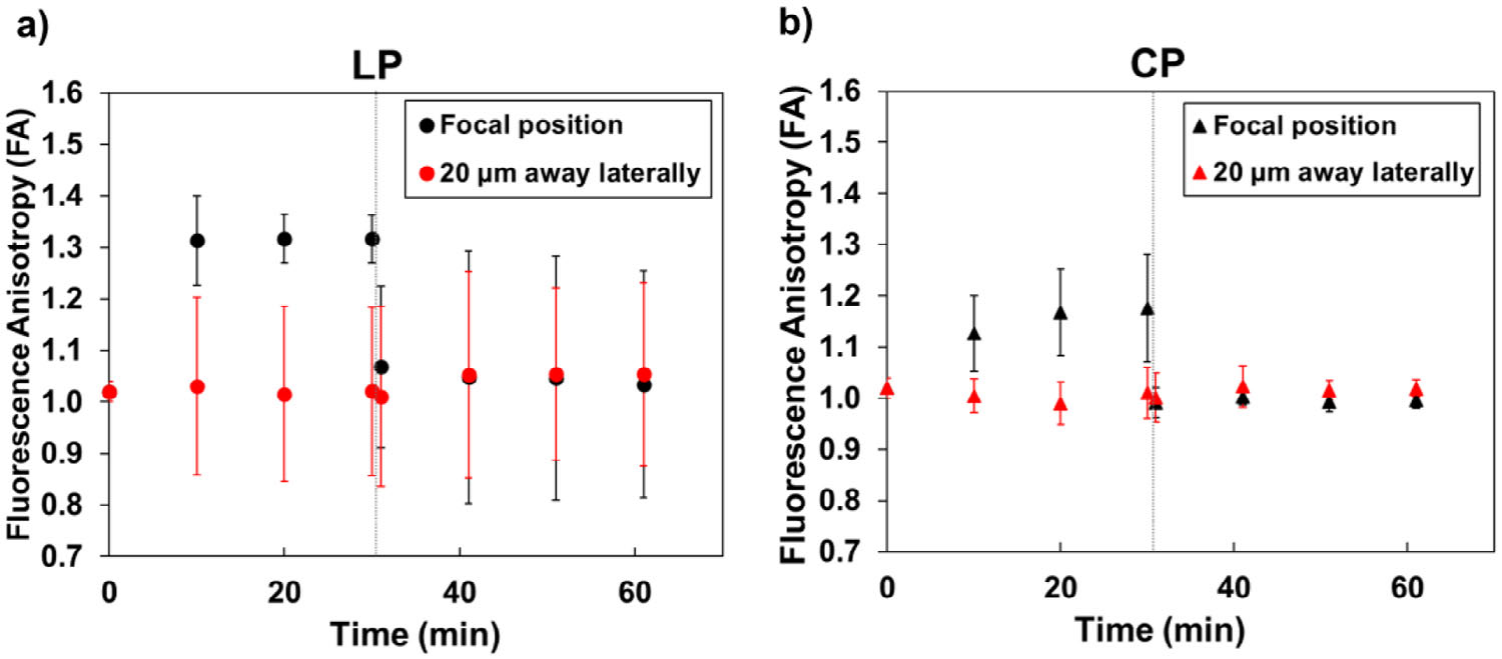
Temporal changes in the fluorescence anisotropy (FA) values under a) LP and b) CP irradiation conditions. Black and red symbols represent the FA at the focal position and 20 µm away laterally, respectively. Error bars indicate the standard deviations from six independent experiments performed with 6 samples.

Figure 4a,b show the averaged FA values with the standard deviations (SDs) for six experiments using each laser polarization mode. The temporal changes of the FA were measured for 60 min:30 min of trapping laser irradiation and 30 min after stopping the laser irradiation. Figure 2 shows that the RFI after 30 min of laser irradiation reaches ≈8, which corresponds with the experimental conditions where the laser polarization dependence of the HEWL crystallization probability is prominently observed, as shown in Figure 3. Figures 2 and 4 clearly show that the FA at the focal point becomes approximately the same value as at 20 µm due to the rapid diffusion of the condensate from the focal position. This indicates that the FA at the focal point after switching off the laser reflects the orientation properties of the HCD, similar to those observed at 20 µm.

When the LP trapping laser was irradiated, the average FA value at the focal point exceeded 1.3 at 10 min of laser irradiation (black circles, Figure 4a). This indicated that the anisotropy of the condensate increased. Although there was no significant change in the average FA value, even with continued laser irradiation, the SDs of the FA values decreased with irradiation time. The observation that FA values exceed 1 suggests that the fluorescent dyes attached to F‐HEWL within the condensate preferentially orient themselves mainly perpendicular to the polarization of the LP trapping laser. Additionally, the decrease in SDs indicates that the orientation of the fluorescent dyes becomes more organized within the condensate.

Conversely, when the CP trapping laser was irradiated, the FA at the focal point showed a different change compared with that using the LP laser. After 10 min of CP laser irradiation, the average FA value at the focal point reached ≈1.1 (black triangles, Figure 4b). Furthermore, with continued laser irradiation, this value slightly increased, reaching ≈1.2 at 30 min. These results indicated that the anisotropy of the condensate increased when the CP laser was irradiated, though not as much as with the LP laser. The cause of this phenomenon is not yet clear; however, it is speculated that the HEWL molecules may align to some extent because the HEWL is a chiral molecule with a secondary structure like alpha‐helices, even when using the CP trapping laser. However, contrary to that of the LP trapping laser, the SD of the FA value during 30 min of CP trapping laser irradiation gradually increased. These results suggest that although the anisotropy of the condensate increases slightly with laser irradiation, the orientation of the fluorescent dyes in the condensate becomes more randomly distributed around their average orientation.

As mentioned earlier, when laser irradiation ceased, the condensate rapidly diffused from the focal point (Figure 1c). After drifting, the condensate gradually settled to the bottom and eventually deposited on the cover glass. We confirmed that the condensate remained in this state without changing into HEWL crystals. This result strongly suggests that the condensate formed at the focal point has no contribution to the crystallization probability shown in Figure 3. Therefore, the results of the temporal changes of the FA of the HCD (outside the focal point) during and after trapping laser irradiation should be considered to elucidate the mechanism of the HEWL crystallization depending on laser polarization.

Figure 4 includes the experimental results of the temporal changes of the FA at 20 µm from the focal point during and after trapping laser irradiation (red circles and red triangles). Although this distance is significantly larger than the focal spot size (≈1 µm), the measurement position is well within the HCD. Figure 4 shows that the average FA during 30 min of laser irradiation is almost 1, regardless of the laser polarization modes. At first glance, this result seems to indicate that the anisotropy of the HCD disappears at a distance from the focal point. However, the dependence of the SDs of the FA values on the polarization mode is noteworthy. The SD of FA is a crucial parameter for evaluating the anisotropy of the HCD domain because it reflects the distribution of molecular orientations within the HCD. A large SD indicates a wide variety of orientations between the six separate experiments, even if the average FA is close to 1, suggesting significant anisotropy within the HCD.

In the case of the LP trapping laser, although the average FA at 20 µm away from the focal spot was close to 1, the HCD itself exhibited a large deviation from FA 1.0 (Figure S6) and a large SD of the FA values, indicating that each HCD was strongly anisotropic. This result also suggests that while each HCD shows strong anisotropy, the orientation of the dye and HEWL molecules within the HCD varied considerably between different samples. This means that each sample exhibited its preferred orientation. However, this orientation differed from sample to sample, as illustrated in Figure S7a. The molecular orientation within the HCD may contribute to the enhanced crystallization observed under LP trapping. In contrast, when using the CP trapping laser, the average FA at 20 µm from the focal spot was also close to 1, and each deviation from FA 1.0 was smaller compared to those with the LP trapping laser (Figure S6). This indicates a smaller SD of the FA values and an equally disordered distribution of orientations among the different samples under CP irradiation (Figure S7b), which may explain the lower crystallization probability observed in this case. This indicates that with CP trapping laser irradiation, the anisotropy of the HCD outside the focal point is minimal. Importantly, this trend was observed both during and after laser irradiation, indicating that the anisotropy of the HCD is maintained even after the laser is turned off. To further confirm the anisotropy of the condensate and HCD induced by optical trapping, we conducted polarized Raman spectroscopy measurements. This technique provides complementary information about molecular orientation and anisotropy, especially for nonfluorescent proteins.^[^ ^17^ ^]^ The detailed results and discussion of the polarized Raman spectroscopy measurements are provided in the Supporting Information (SI 7). The polarized Raman spectroscopy results fully support the observations obtained from the FA measurements, confirming the presence of anisotropy in the HCD and its dependence on the laser polarization mode.

### Mechanism of Laser Polarization‐Dependent Crystallization Behavior

The results of the anisotropy of the condensate (focal spot) and the HCD (outside), inferred from the FA measurement results presented in Figure 4, are summarized in Table 1.

**Table 1:** Summary of the anisotropy of the condensate and the HCD induced by different laser polarization modes. (S: strong, M: moderate, W: weak)

Point	Polarization Modes	30 min Irradiation	30 min Post‐Irradiation
		Anisotropy	Anisotropy
Focal spot	LP	S	–
	CP	S	–
Outside (20 µm)	LP	M	M
	CP	W	W

**Figure 5:**
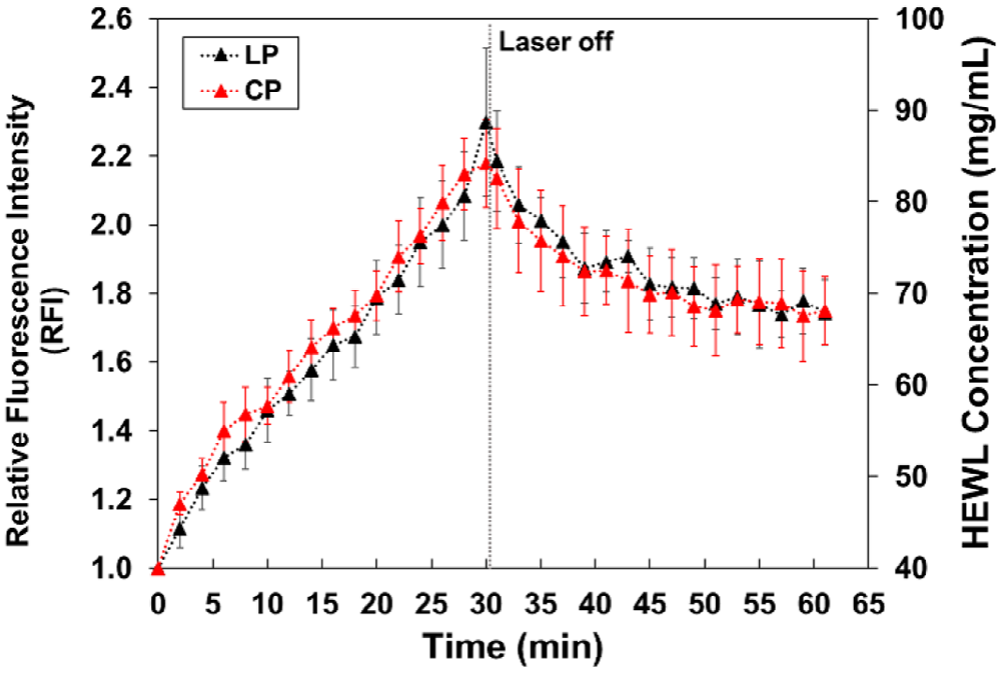
Temporal changes in the RFI and HEWL concentration at 20 µm from the focal point during and after laser irradiation. Black and red dots represent the temporal changes in the RFI under LP and CP irradiation conditions, respectively. Error bars represent standard deviation from three independent experiments.

When discussing the crystallization mechanism, the anisotropy of the HCD at 20 µm from the focal point is more critical than that at the focal point. To further investigate the concentration dynamics outside the focal point, we analyzed the temporal changes in HEWL concentration at 20 µm from the focal point. This analysis is crucial for understanding the formation and behavior of the HCD, which plays a significant role in the crystallization process. Figure 5 illustrates the temporal changes in HEWL concentration at this position under the same experimental conditions as in Figure 2.

Similar to the observations at the focal point (Figure 2), the concentration of HEWL increased almost linearly with the trapping laser irradiation time, irrespective of the laser polarization modes. However, the concentration reached ≈2.2 times the initial concentration, which is lower than that observed at the focal point. After the laser irradiation was stopped, the HEWL concentration gradually decreased, reaching ≈1.8 times the initial concentration after 30 min. Notably, it took more than 2 h for the concentration to return to its initial state, indicating slow diffusion of HEWL molecules within the HCD.

Such an extremely slow diffusion rate of the HEWL molecules in the HCD generated by optical trapping has been reported in some previous studies.^[^ ^15^, ^16^, ^18^ ^]^ These findings suggest that the crystallization probability dependent on laser polarization is not simply caused by concentration increases but primarily by the anisotropy of the HCD.

**Figure 6:**
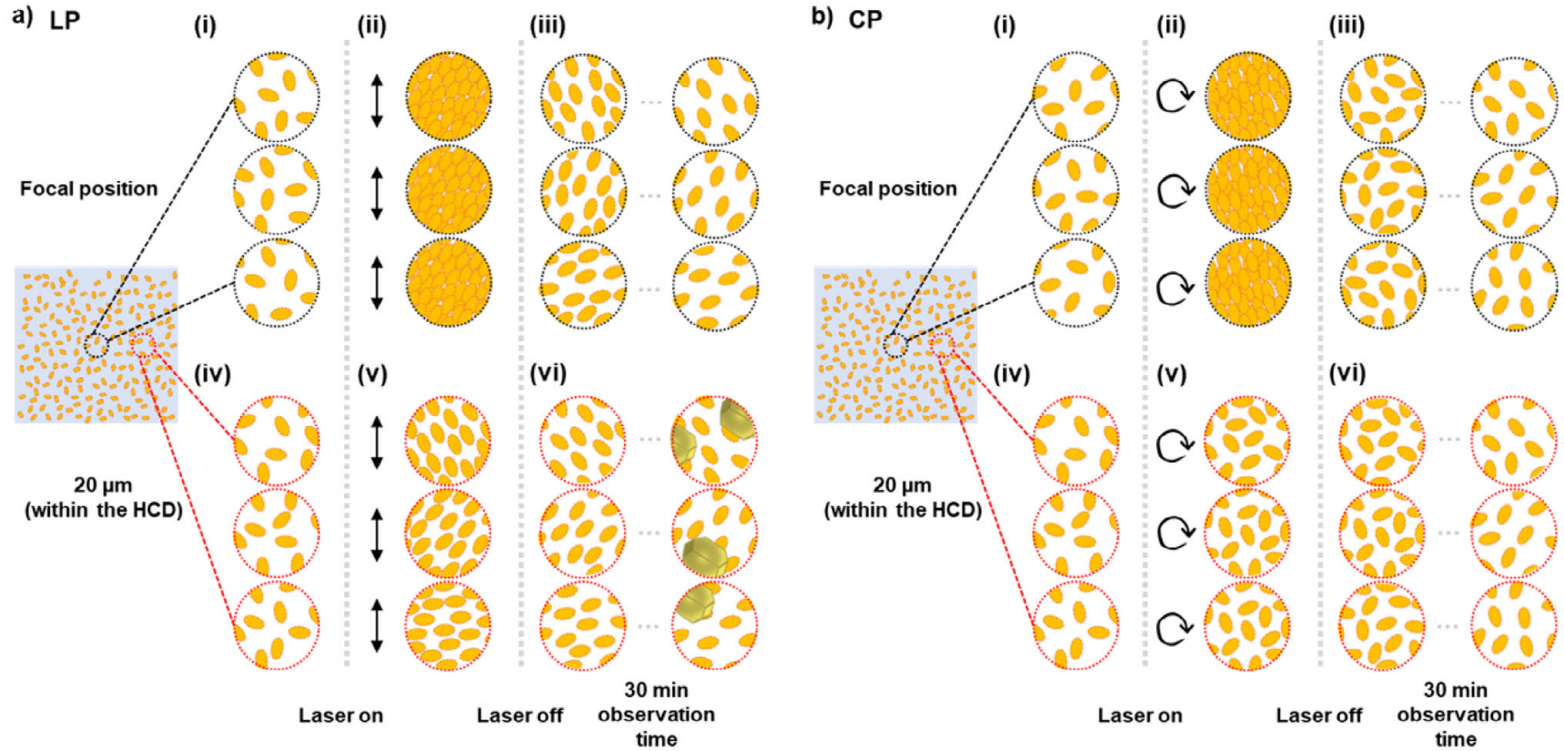
Schematic illustration of laser polarization‐dependent dynamics of HEWL molecular anisotropy within the condensate and the HCD. The diagram depicts the concentration dynamics and anisotropy changes of the condensate and the HCD before, during, and after a) LP irradiation and b) CP irradiation. Panels (i)–(iii) and (iv)–(vi) illustrate the dynamics of HEWL molecules at the focal position and 20 µm away from the focal position (within the HCD), respectively: (i) and (iv) before laser irradiation, (ii) and (v) during laser irradiation, and (iii) and (vi) after laser irradiation. The three vertically arranged illustrations in each panel represent the conditions of different samples.

Based on the results presented in Table 1 and Figure 5, we discuss the dynamics of the HCD formation and the crystallization mechanism influenced by laser polarization modes, as illustrated in Figure 6.

Upon the initiation of laser irradiation with LP and CP, the HEWL molecules/clusters are trapped at the focal point by the gradient force of the focused laser beam, increasing the local concentration and forming the condensate. Concurrently, the concentration in the vicinity of the focal point increased, forming the HCD around the focal spot (Figure 6a(ii,v),b(ii,v)). This HCD is likely formed as the condensate consumed the surrounding HEWL molecules/clusters during laser irradiation. The condensate's stability, provided by optical trapping, facilitated this process.

The anisotropy of the condensate and the HCD is influenced by the laser polarization modes. Under LP trapping laser irradiation, as shown in Figure 6a(ii,v), the anisotropy of the condensate and the HCD significantly increases with the laser irradiation, while the orientation of the HEWL molecules within the HCD differs in each trial. We hypothesize that laser‐induced physical phenomena, such as photo‐induced orientation^[^ ^19^, ^20^, ^21^, ^22^, ^23^ ^]^ and optical Kerr effect,^[^ ^24^, ^25^, ^26^, ^27^ ^]^ contribute to the observed increase in the anisotropy within the condensate. Photo‐induced orientation occurs when molecules interact with polarized light and align themselves along the polarization direction. In our study, the LP trapping laser interacts with HEWL molecules, causing them to align along the direction of laser polarization. This alignment could lead to strong anisotropy observed in the condensate. While the optical Kerr effect is typically observed with pulsed lasers, some studies have reported its occurrence with continuous‐wave lasers, particularly at high photon densities (7 MW cm^−2^ and 2.6 W cm^−12^).^[^ ^24^, ^28^ ^]^ Notably, the photon density (60 MW cm^−2^) employed in our study significantly exceeds that used in these previous reports. This suggests that the optical Kerr effect may also play a role in the observed anisotropy despite the use of a continuous‐wave laser. Importantly, the anisotropy of the condensate achieved at the laser focus considerably extends beyond the focal point, as indicated by the moderate anisotropy of the HCD at 20 µm from the focus. The underlying cause of the random orientation of HEWL molecules within the HCD in each trial remains unclear at present. However, our study reveals that it is the increase in anisotropy of the HCD, rather than the specific orientation of HEWL molecules, that contributes significantly to the enhancement of crystallization efficiency. As such, elucidating the mechanisms driving this random orientation and its role in crystallization will be a critical focus of future research. Meanwhile, CP trapping laser irradiation resulted in a comparable anisotropy of the condensate to that of LP trapping at the focal point, and the HCD at 20 µm from the focal point shows weak anisotropy.

As shown in Figure 6a(iii,vi),b(iii,vi), after stopping the laser irradiation, the condensate is migrated, and the concentration and anisotropy of the HCD gradually decrease and slowly return to an isotropic state. Notably, for the LP trapping laser irradiation, the moderate anisotropy of the HCD persists even after the laser irradiation is stopped (Figure 6a(vi) and Table 1). Considering that concentration dynamics (Figure 5) after turning off the trapping laser is almost independent of the laser polarization mode, the difference in the HCD anisotropy at 30 min post‐irradiation is considered to cause a strong dependence of the crystallization probability on laser polarization.

According to a two‐step nucleation model,^[^ ^5^, ^6^, ^7^ ^]^ unlike classical nucleation theory, where concentration increases and molecular orientation progresses simultaneously, a liquid droplet is first formed by surpassing the first activation energy. Thereafter, the molecular orientation within the droplet surpasses the second activation energy, leading to crystallization. Based on this model, we propose the following mechanism: optical trapping generates a highly anisotropic condensate (droplet) at the focal point. This condensate, exceptionally stable under optical trapping, grows by adsorbing surrounding HEWL molecules/clusters, forming the HCD. The anisotropic nature of this HCD confers significant stability, inhibiting crystallization during laser irradiation. Upon the cessation of laser irradiation, the HEWL clusters within the HCD gradually diffuse to the surroundings, with a decrease in the overall anisotropy of the HCD. This process results in the formation of an HCD with a moderately high concentration and moderate anisotropy, which is precisely the condition that promotes HEWL crystallization.

## Conclusion

This study highlights the crucial role of laser polarization in controlling HEWL crystallization and provides a powerful approach for manipulating the dynamics of this process. Modulating HEWL molecule orientation via laser polarization offers new possibilities for protein crystallization techniques. Specifically, LP trapping laser irradiation induced a high degree of anisotropy, significantly enhancing crystallization probability. Conversely, CP trapping laser irradiation afforded low anisotropy, leading to minimal impact on crystallization. These results underscore the critical role of HEWL molecule orientation in the crystallization process under optical trapping conditions. By manipulating anisotropy through laser polarization modes, we can effectively control the crystallization dynamics of HEWL. A comprehensive discussion on the role of anisotropy in protein crystals has been provided.^[^ ^29^ ^]^ This newfound understanding opens exciting avenues for the development of crystallization techniques applicable to HEWL and a wide array of proteins and biomolecules. A comprehensive discussion has been provided for future perspectives on optical trapping and protein crystallization.^[^ ^30^, ^31^ ^]^ Furthermore, the findings from this study have the potential to transform current protein crystallization methods. The ability to control anisotropy precisely using laser polarization modes could lead to the development of new crystallization processes or significantly enhance the efficiency and yield of current methods. This advancement holds immense promise to produce high‐quality protein crystals, which are essential for structural biology research and drug discovery in the biotechnology and pharmaceutical industries.

## Supporting Information

The authors have cited additional references within the Supporting Information.^[^ ^32^, ^33^, ^34^, ^35^, ^36^, ^37^, ^38^, ^39^, ^40^ ^]^

## Conflict of Interests

The authors declare no conflict of interest.

## Supporting information



Supporting Information

## Data Availability

The data that support the findings of this study are available from the corresponding author upon reasonable request.
