# Supplementary material for: Laser‐Polarization‐Induced Anisotropy Enhances Protein Crystallization
Source: Angew Chem Int Ed Engl. 2025 Mar 17;64(21):e202501827. doi: 10.1002/anie.202501827 (PMC12087825; doi:10.1002/anie.202501827)
Supplement: Supplementary file 1 — Supporting Information [file ANIE-64-e202501827-s001.docx]

*Supporting Information*

Laser-Polarization-Induced Anisotropy Enhances Protein Crystallization

Tien Chen^†^, Hirotsugu Hiramatsu^†^, Shuichi Toyouchi^‡^, and Teruki Sugiyama^†,§*^

^†^Department of Applied Chemistry and Center for Emergent Functional Matter Science, National Yang Ming Chiao Tung University, Hsinchu 300093, Taiwan

^‡^Research Institute for Light-induced Acceleration System (RILACS), Osaka Metropolitan University, Sakai, Osaka 599-8570, Japan

^§^Division of Materials Science, Nara Institute of Science and Technology, 8916-5 Takayama-cho, Ikoma, Nara 630-0192, Japan

**Table of Contents**

**Methods and Materials**  [S3](#_heading=h.3znysh7)

[**SI 1. Theoretical Basis for Optical Trapping of HEWL**  S6](#_heading=h.3znysh7)

[**SI 2. Calibration Curve for Fluorescence Intensity Ratio and HEWL Concentration**](#_heading=h.2et92p0)   [S8](#_heading=h.2et92p0)

[**SI 3. Definition of RFI_90_ and RFI_00_ under Different Polarized Conditions**](#_heading=h.2et92p0)  S9

**[SI 4.](#_heading=h.3dy6vkm)** **[Estimation of FA under Different HEWL Concentrations and its Calculation](#_heading=h.3dy6vkm)**  [S](#_heading=h.3dy6vkm)10

**SI 5.** **Distribution of FA for HEWL Crystallization under LP and CP Laser Irradiation**  S11

[**SI 6.** **Assumptions Regarding SDs for Distinguishing Isotropy and Anisotropy**  S](#_heading=h.3dy6vkm)12

[**SI 7.** **Investigating Anisotropy of Condensate and High-concentration Domain Induced by Optical Trapping Using Polarized Raman Spectroscopy**  S](#_heading=h.3dy6vkm)13

[**References**  S](#_heading=h.3dy6vkm)17

**Methods and Materials**

**HEWL Sample Preparation**

Throughout all stages of hen egg-white lysozyme (HEWL) sample preparation, D_2_O (Sigma-Aldrich, 99.9 atom %D) was utilized as the solvent instead of H_2_O. This substitution minimizes the photon absorption by H_2_O at 1064 nm.^[S1]^ Previous reports have successfully estimated the local temperature elevation at the focal point under optical trapping conditions to be 22-24 K/W for H_2_O and 2-3 K/W for D_2_O.^[S2,3]^ According to these reports, we estimated the local temperature increase at the focal point for the HEWL/sodium acetate D_2_O buffer solution utilized in this experiment to be approximately 2.0 K/W.^[S4]^

An acetate buffer solution (100 mM, pD = 5.1) was used as a solvent. The commercial HEWL powder (> 99.9%) purchased from FUJIFILM Wako was then dissolved in the acetate buffer solution to prepare the HEWL mother solution (80 mg/mL). Immediately before the optical trapping experiments started, the HEWL mother solution was promptly mixed with NaCl D_2_O solution (5.4% w/v) to prepare the sample solution containing HEWL at 40 mg/mL and NaCl at 2.7% w/v. For experiments on optical trapping and polarized Raman measurement, 20 μL of the HEWL sample solution was poured into a home-made container (Figure S1), resulting in a solution thickness of approximately 110-130 μm. The container was subsequently sealed with another cover glass to minimize solvent evaporation.


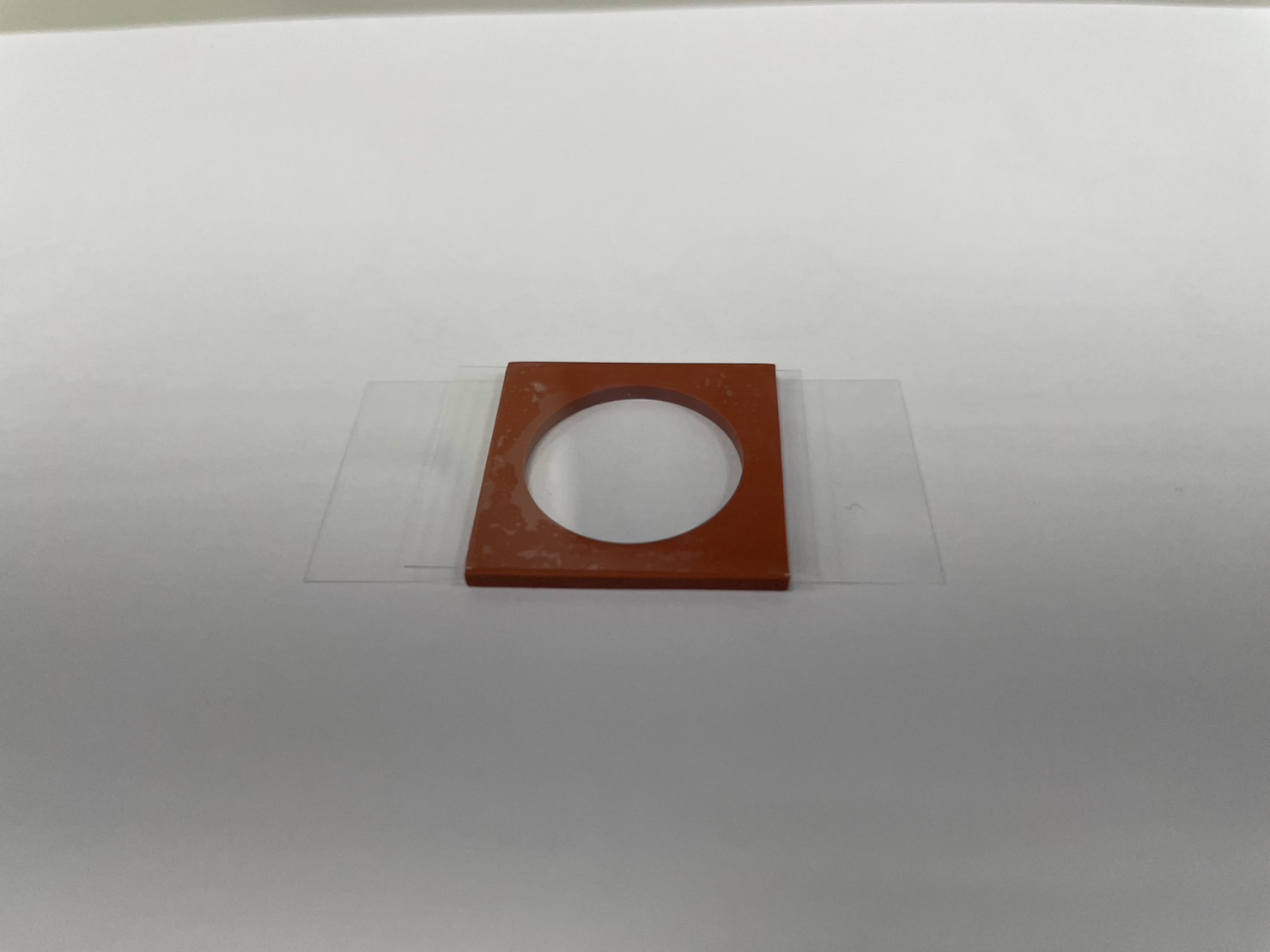


**Figure S1.** Photograph of the custom-made silicone container used in this experiment.

Fluorescent dye-labeled HEWL (F-HEWL) was utilized to examine the temporal change in the concentration of HEWL at/around the focal point during the laser irradiation. The F-HEWL preparation followed the previously described protocol.^[S4]^ In this experiment, the HEWL mother solution (105 mg/mL), NaCl D_2_O solution (5.4%(w/v)), and F-HEWL (0.05 mg/mL) were mixed in a volume ratio of 8:8:5 immediately before starting the laser irradiation. Then, 20 μL of the sample solution with the solution conditions (HEWL: 40 mg/mL; NaCl: 2.7% (w/v); F-HEWL: 0.01 mg/mL) was dropped into the home-made container.

**Optical Setup for Optical Trapping Experiments and Fluorescence Measurement**


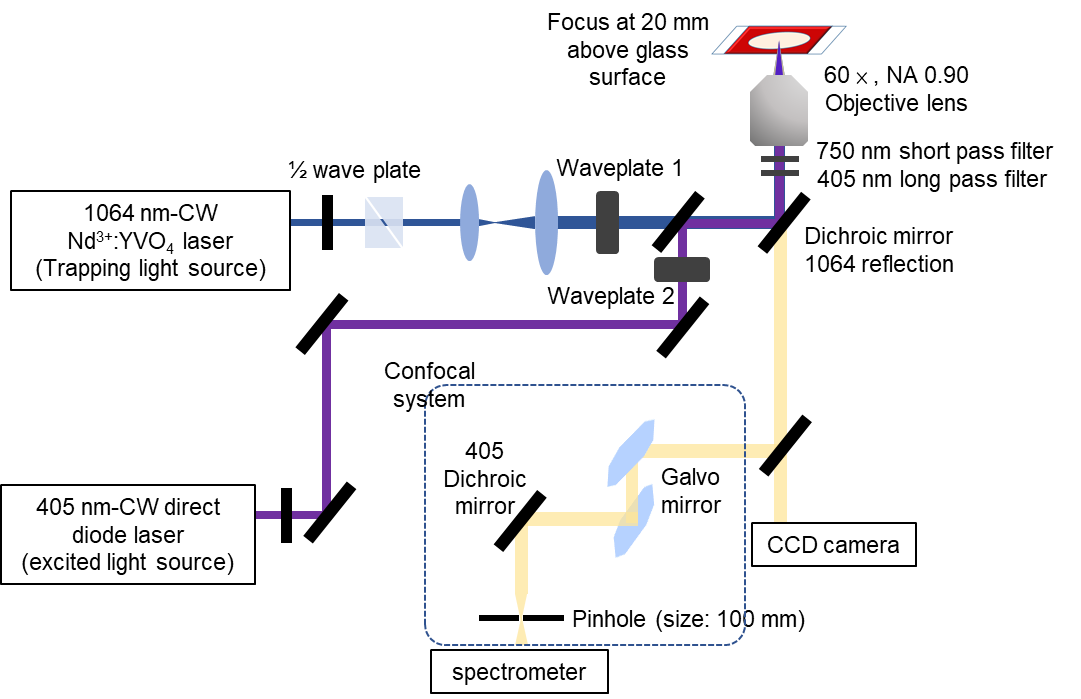
Figure S2 shows the optical setup of the optical trapping experiment and fluorescence measurement. A continuous-wave (CW) laser with a wavelength of 1064 nm from a Nd^3+^:YVO_4_ laser (Spectra-Physics, J201-BL-106C) was introduced into an inverted microscope (Olympus, IX71) as the trapping light source and was tightly focused through an objective lens (Olympus, 60×, NA 0.9) into the sample solution. The power of the trapping laser was set to 1.0 W after passing through the objective lens. The half-wave and quarter-wave plates were used to adjust the polarization modes of the trapping laser.
 For fluorescence measurements, a CW diode violet laser with a wavelength of 405 nm (Spectra-Physics, EXLSR-405C-100-CDRH) was used as the excitation light source. The laser power of the 405 nm laser was fixed at 10 μW after the objective lens and focused into the focal spot as the trapping laser. To manipulate the polarization modes of the excitation laser, either a half-waveplate or a quarter-waveplate was employed. These plates were used to either adjust the polarization direction of the 405 nm LP or convert it to 405 nm CP. Short-pass (< 750 nm) and long-pass (> 405 nm) filters were placed inside the microscope to block the reflected light of both laser beams. The fluorescence emission from the F-HEWL was detected using a charge-coupled device (CCD) camera (PIXIS: 400B) at 198.5 K, equipped with a spectrometer (Princeton Instruments, SP2300i) through a 150 μm pinhole.

Figure S2. Optical setup for optical trapping and simultaneous fluorescence measurement.

**Polarized Raman Measurement**

The optical setup of polarized Raman measurement is shown in Figure S3. A linearly polarized CW laser with a wavelength of 532 nm (Spectra-Physics, Millennia Pro s-Series, 10sJS) was used as the excitation light source and was introduced into the inverted microscope via the same path as the trapping laser. After passing through the objective lens, the laser power was consistently fixed at 20 mW. The Rayleigh scattering of the 1064 nm trapping laser and the 532 nm excitation laser were removed by a notch filter (Semrock, NF03-532/1064E-25). The Raman signals through a 100 μm pinhole before the polarizing beam splitter were separated into horizontally and vertically polarized light to measure the Raman polarization ratio. The polarized Raman signals were detected at 188 K using a CCD camera (Andor, DU401A-BVF) device and a spectrometer (Andor, SR-303i-A). Tetrachloromethane (CCl_4_), a symmetric compound, was used as the standard sample to calibrate the detector. According to the principles of polarized Raman spectroscopy, the depolarization ratio (DR) should be 0.75 for an asymmetric vibration mode.^[S5]^ We monitored the Raman signal of the asymmetric stretching (*v*_3_) mode of CCl_4_ as the standard.^[S6]^ The detected DR of *v*_3_ was found to be 0.83. The discrepancy between the experimental and theoretical values was attributed to the instrument setup. Subsequently, a correction coefficient was calculated based on the ratio of the theoretical to the experimental values. All depolarization values were then calibrated using this correction coefficient.


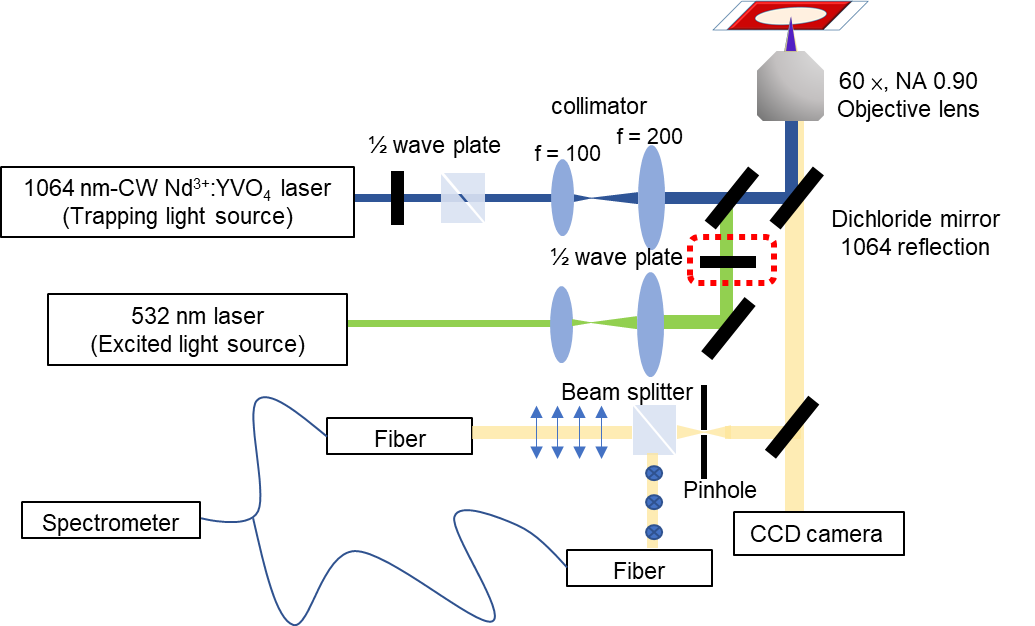


Figure S3. Optical setup for optical trapping and simultaneous polarized Raman measurement.

**SI 1.** **Theoretical Basis for Optical Trapping of HEWL**

HEWL has a propensity to self-associate and form clusters in solution, particularly at higher concentrations.^[S7, S8]^ These HEWL clusters, more significant than individual HEWL molecules, are more effectively trapped by optical trapping due to the increased gradient force acting on them. Therefore, we employ optical trapping in this study to manipulate HEWL molecules/clusters. It is plausible to assume that their size is smaller than the wavelength of the trapping laser (1 μm). According to Rayleigh scattering theory, the cluster can be treated as a single electric dipole, and the optical force exerted on one electric dipole from the surrounding electric field can be calculated as the Lorentz force.^[S9]^ Assuming that the HEWL clusters have negligible absorption at the laser wavelength of 1064 nm, the optical Lorentz force (***F***_opt_) can be expressed as the sum of two optical forces (Eq. S1): the gradient force (***F****_grad_*) acting along the laser intensity gradient and the scattering force (***F****_scat_*) acting in the direction of the Poynting vector.

$\boldsymbol{F}_{opt}=\boldsymbol{F}_{grad}+\boldsymbol{F}_{scat}=\frac{1}{2}\alpha\nabla\boldsymbol{E}^{2}+\alpha\frac{\partial}{\partial t}(\boldsymbol{E}\times\boldsymbol{B})$…(Eq. S1),

where ***E*** and ***B*** represent the electric field and magnetic flux density, respectively. $\nabla$ indicates the gradient of spatial coordinates, and *α* denotes the polarizability of the cluster, as described by the following Eq. S2,

$\alpha=4\pi\varepsilon_{0}\varepsilon_{m}r^{3}\frac{{(\frac{n_{t}}{n_{m}})}^{2}-1}{{(\frac{n_{t}}{n_{m}})}^{2}+2}$…(Eq. S2),

where *r* represents the radius of the cluster, *n_t_* and *n_m_* stand for the refractive indices of the cluster and the surrounding medium, respectively, and *ε_0_* and *ε_m_* denote the permittivity of vacuum and the relative permittivity of the surrounding medium, respectively. When the trapping laser is tightly focused using a high-NA objective lens, the influence of ***F_grad_*** becomes significantly more pronounced than that of ***F_scat_***. Consequently, the resultant trapping force can be reasonably approximated using the Eq. S3 provided below:

$\boldsymbol{F} \sim\boldsymbol{F}_{grad}=\frac{1}{2}\alpha\nabla\boldsymbol{E}^{2}$…(Eq. S3)

Eq. S3 indicates that ***F_grad_*** is directly proportional to the power of the employed trapping laser and the volume of the cluster. Furthermore, the light-cluster interactions lead to the formation of an optical potential well characterized by a potential energy (*U*) described by Eq. S4 below:

$U= -\int\boldsymbol{F}_{grad}dr=-\frac{1}{2}\alpha\boldsymbol{E}^{2}$…(Eq. S4)

When the optical potential energy *U* overcomes the thermal kinetic energy (kT) of clusters in the solution, ^[S10]^ trapping of these clusters becomes Our calculations, taking into account the experimental parameters and assuming a cluster refractive index of 1.45, suggest that optical trapping can occur for clusters with a radius of approximately 130 nm or more significant, where *U* equals the thermal energy kT. The optical force generated in our current experiment might initially appear too weak to establish stable trapping of the clusters, particularly in the early stages of laser irradiation. However, once the interaction between light and clusters creates an optical potential well, the gradient force experienced by subsequent clusters entering the focal region becomes more pronounced due to the increased refractive index at the laser focus.

**SI 2.** **Calibration Curve for Fluorescence Intensity Ratio and HEWL Concentration**

This study utilized the fluorescence intensity ratio to estimate the HEWL concentration. The calibration curve was done by monitoring the fluorescence intensity of the F-HEWL/HEWL mixture with various concentrations. The recipe for the mixture is shown in Table S1. The molar ratio between HEWL and F-HEWL was fixed at 3360:1 in all the mixtures. The relative fluorescence intensity (RFI) was calculated relative to the fluorescence intensity of the initial solution before laser irradiation (40 mg/mL HEWL, 0.01 mg/mL F-HEWL). The calibration line between the relative fluorescence intensity and HEWL concentration is shown in Figure S4. From the results, the concentration of HEWL is proportional to the relative fluorescence intensity. Furthermore, we confirmed that 9 times the F-HEWL concentration did not cause the saturation of fluorescence intensity.

Table S1. Concentration of HEWL and F-HEWL in different mixtures.

| Mixtures number | 1 | 2 | 3 | 4 | 5 | 6 | 7 | 8 | 9 |
| --- | --- | --- | --- | --- | --- | --- | --- | --- | --- |
| HEWL concentration (mg/mL) | 40 | 80 | 120 | 160 | 200 | 240 | 280 | 320 | 360 |
| F-HEWL  concentration (mg/mL) | 0.01 | 0.02 | 0.03 | 0.04 | 0.05 | 0.06 | 0.07 | 0.08 | 0.09 |


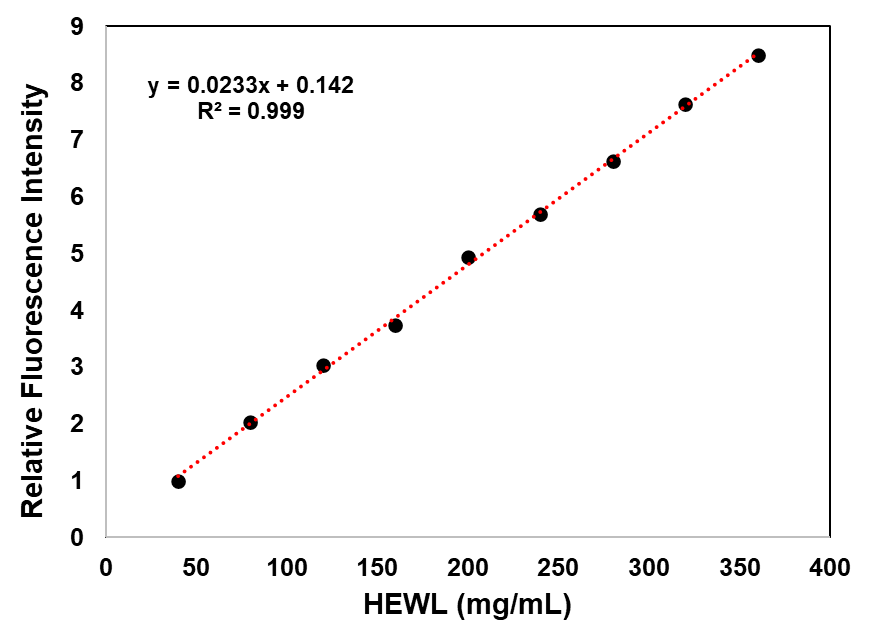


**Figure S4**. Calibration curve showing the relationship between relative fluorescence intensity and HEWL

concentration.

**SI 3. Definition of RFI_90_ and RFI_00_ under Different Polarized Conditions**

**
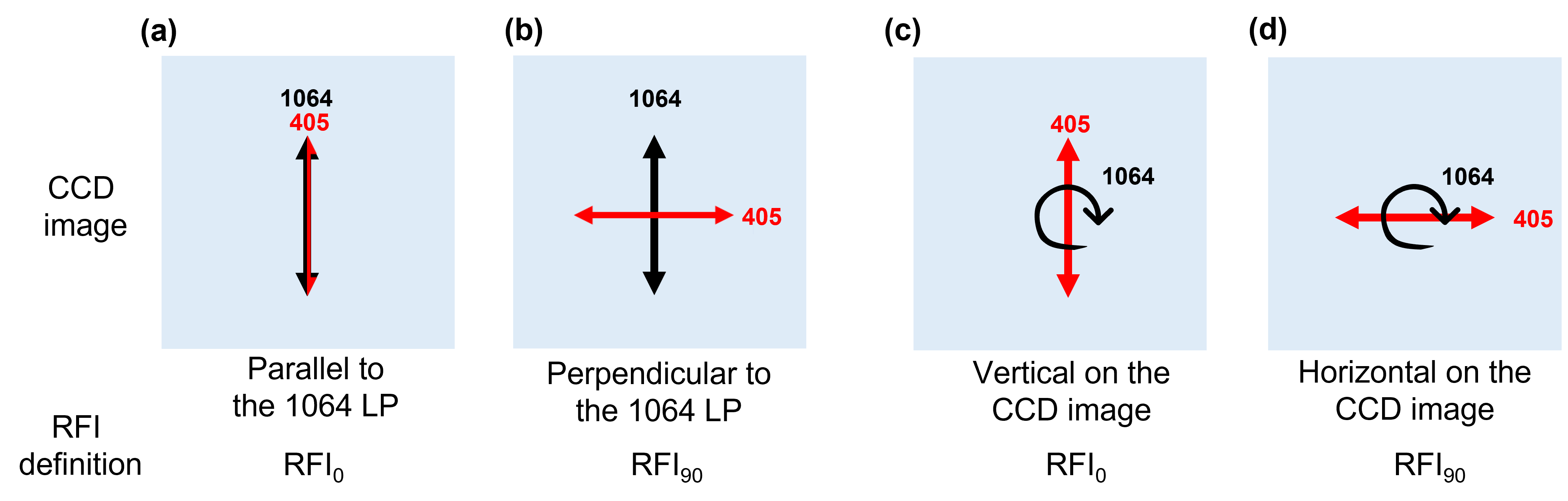
**

**Figure S5**. Scheme illustrating the definitions of RFI_90_ and RFI_0_. Under the 1064 LP condition: (a) RFI_0_ is defined when the polarization direction of 405 LP is parallel to the 1064 LP direction, and (b) RFI_90_ is defined when the polarization direction of 405 LP is perpendicular to the 1064 LP direction. Under the 1064 CP condition, (c) RFI_0_ is defined when the polarization direction of 405 LP is vertical on the CCD image, and (d) RFI_90_ is defined when the polarization direction of 405 LP is horizontal on the CCD image.

SI 4. Estimation of FA under Different HEWL Concentrations and its Calculation

**Table S2.** FA under different HEWL concentrations.

| **HEWL concentration (mg/mL)** | **FA** |
| --- | --- |
| 8 | 1.04 ± 0.01 |
| 40 | 1.02 ± 0.02 |

**SI 5.** **Distribution of FA for HEWL Crystallization under LP and CP Laser Irradiation**

**
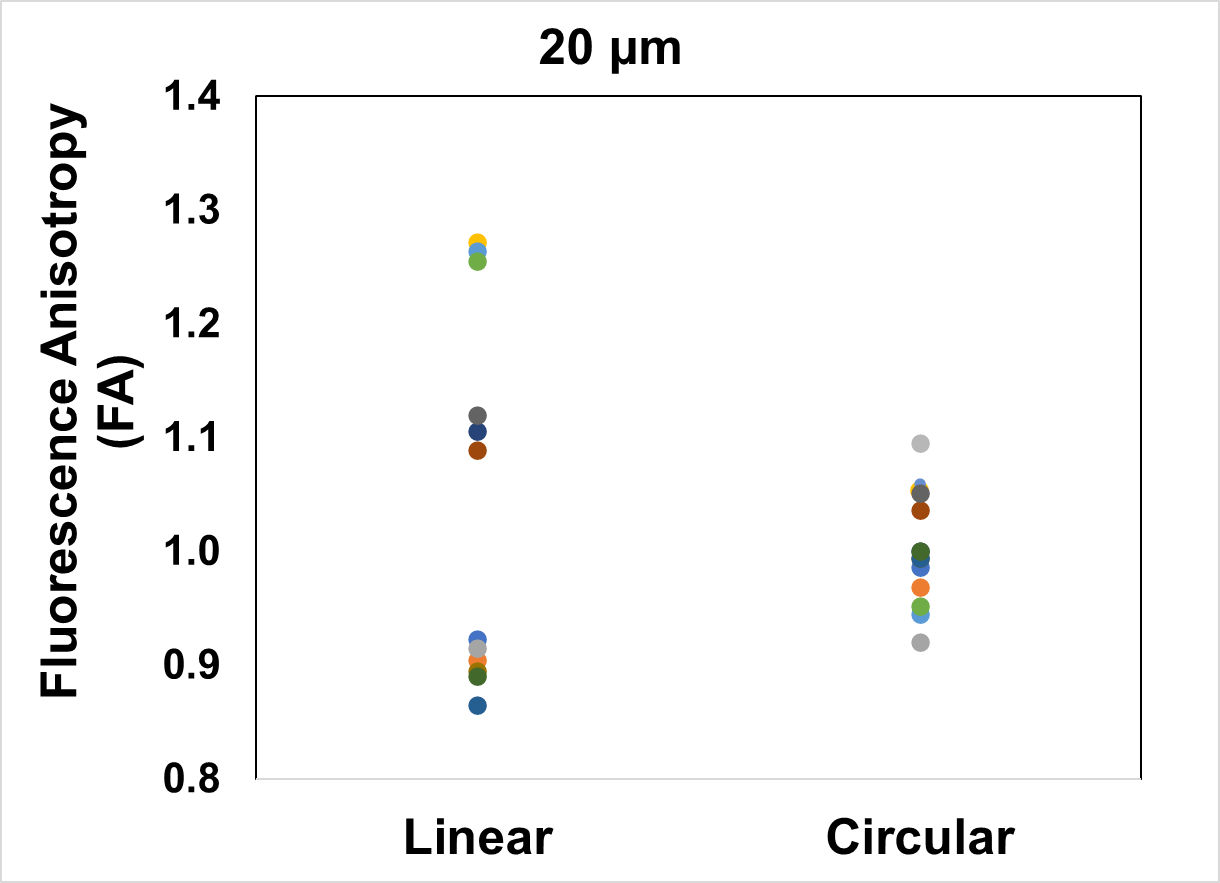
**

**Figure S6.** Distribution of FA for HEWL crystallization under LP and CP laser irradiation.

**SI 6. Assumptions Regarding SDs for Distinguishing Isotropy and Anisotropy**

**
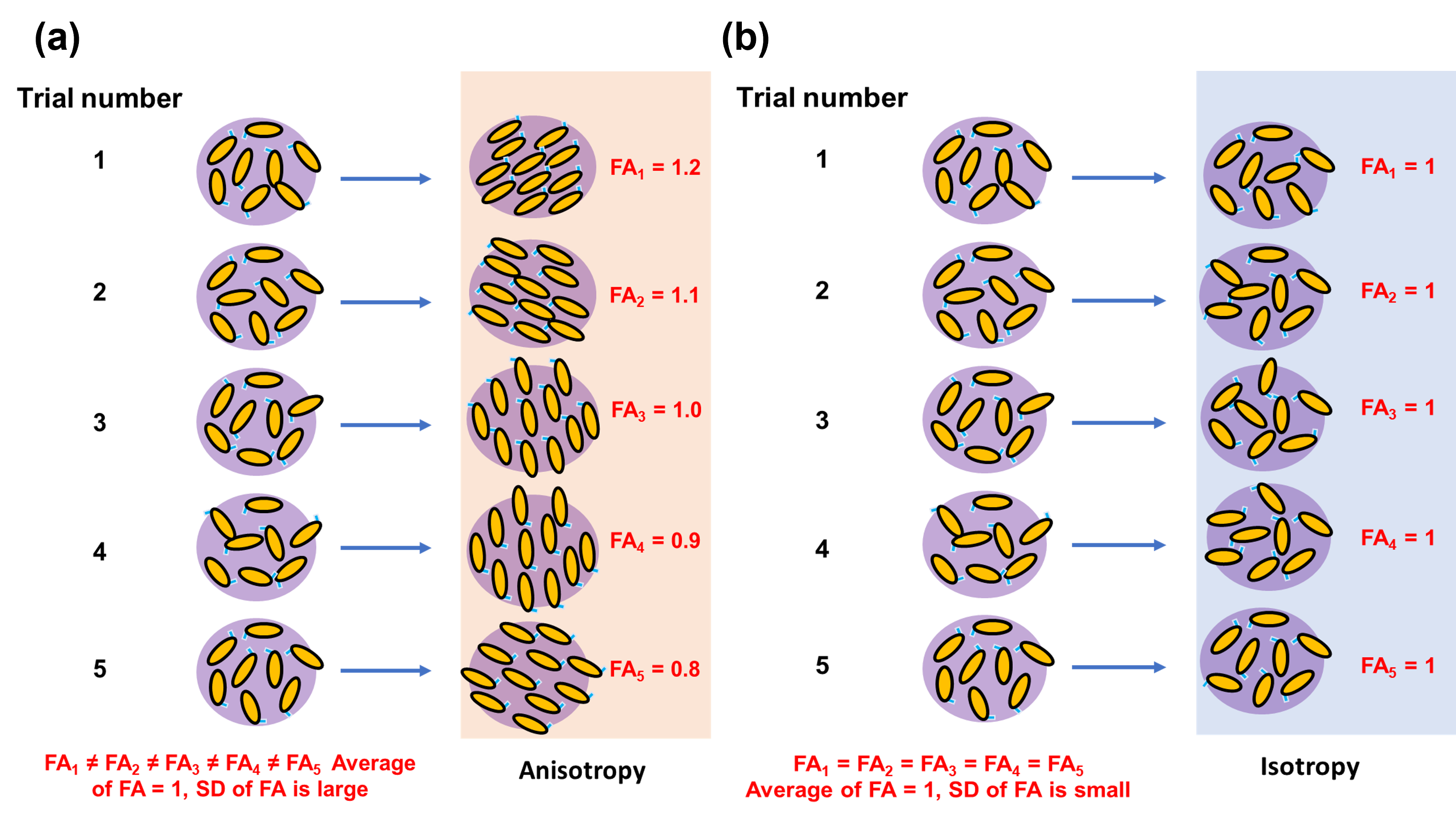
**

**Figure S7.** Illustration of how standard deviation (SD) of fluorescence anisotropy (FA) values can distinguish between isotropic and anisotropic samples, even when the average FA is 1. (a) Anisotropy: Individual FA measurements vary across trials, resulting in a large SD despite the average FA being 1; (b) Isotropy: All individual FA measurements are consistently 1, resulting in a small SD. Trials 1–5 represent independent measurements.

SI 7. Investigating Anisotropy of Condensate and High-concentration Domain Induced by Optical Trapping Using Polarized Raman Spectroscopy

The depolarization ratio (DR) obtained by the polarized Raman spectroscopy is an intrinsic constant for each vibrational mode of isotropically distributed molecules. When the molecular distribution becomes anisotropic, the DR value can deviate from this intrinsic constant. It can complement FA measurements for anisotropy of the condensate and the HCD. Therefore, we employed DR as a probe to assess the anisotropy of the condensate and the HCD. Details of the optical setup are depicted in Figure S3.

This experiment was conducted under the same experimental conditions as that shown in Figure 1, using HEWL buffer solutions without F-HEWL. Polarized Raman spectroscopy measurements were conducted every 5 or 10 min at and 20 μm from the focal point during (30 min: 0–30 min) and after (30 min: 31–61 min) laser irradiation. Further, the temporal changes in the DR values were examined. The typical Raman spectrum of the sample solution is shown in Figure S8, and the DR value was analyzed based on the vibrational mode at 1,453 cm^−1^, including CH, CH_2_, and CH_3_ deformation and scissoring vibrational modes. For molecules mostly with *C_1_* symmetry, such as proteins, the strict distinction between totally symmetric and non-totally symmetric vibrations becomes less applicable. Consequently, the depolarization ratio (DR) can vary across different vibrational bands. Specifically, in the 1400-1500 cm-1 region of interest, multiple CH2 bending vibrational modes overlap. Therefore, the observed DR in this region represents an averaged value across these overlapping bands, rendering the discussion of a singular, theoretically predicted value less meaningful. The DR before trapping laser activation is typically around 0.6 ± 0.2, as shown in Figures S9a and S9b. However, it is crucial to note that the DR changes within the initial five minutes of post-laser irradiation. Based on this observation, we posit that the subtle anisotropy within the solution arises within this immediate five-minute interval following laser irradiation. Repeated experiments were conducted 6–12 times under LP and CP trapping laser irradiation conditions, and the results of the temporal changes in the average DR values at and 20 μm from the focal point are summarized in Figures S9a and S9b, respectively.


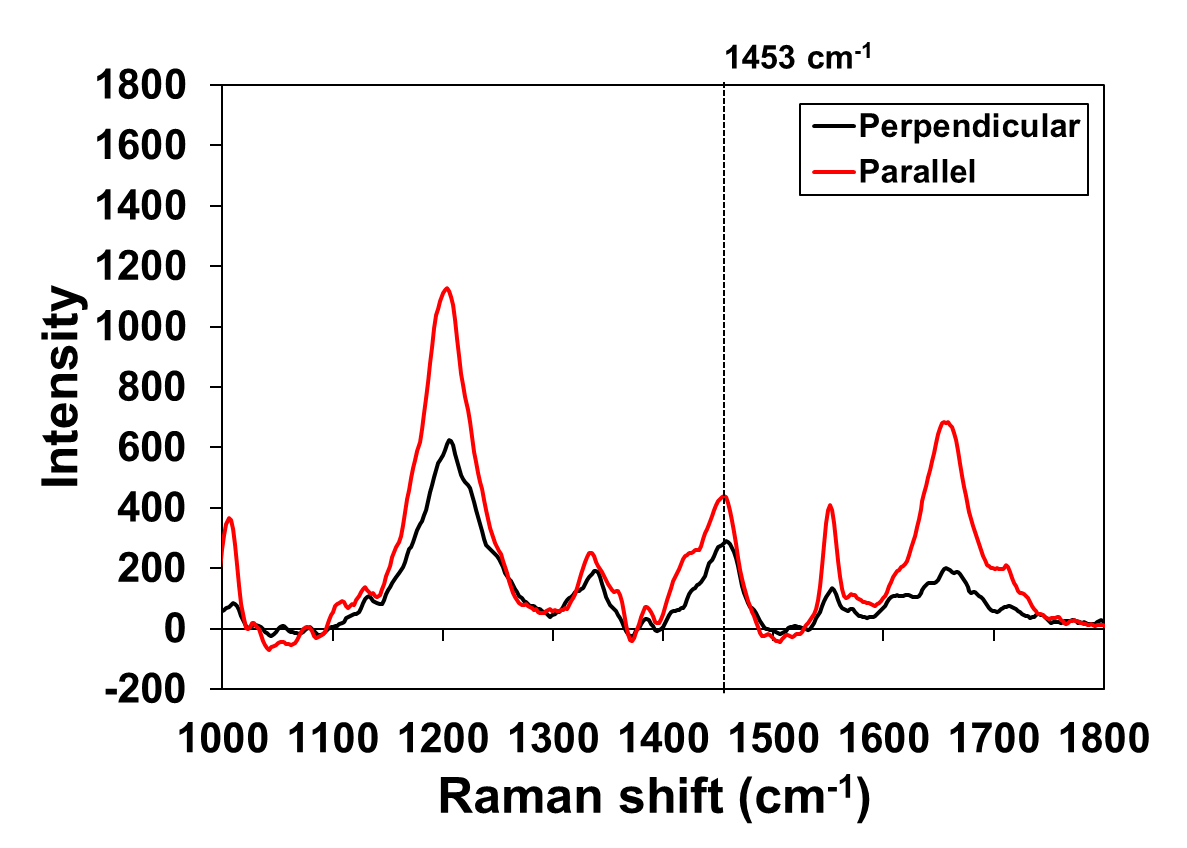


Figure S8. Typical Raman spectra of the HEWL sample solution. The red and black lines represent the Raman signals parallel and perpendicular to the polarization direction of the excitation laser, respectively.

The average DR values at the focal point decreased over the laser irradiation time, regardless of the laser polarization modes (black circles and triangles in Figures S9a and S9b, respectively). These results arise from increased anisotropy of the condensate formed at the focal point and fully support the FA measurement results shown in Figure 4. In contrast, the temporal change in the average DR value at 20 μm away from the focal point (red circles and triangles in Figures S9a and S9b, respectively) was more gradual than that at the focal point. Specifically, the average DR value remained almost constant, maintaining the initial state during and after laser irradiation, regardless of the laser polarization mode. However, to discuss the anisotropy of the HCD, it is essential to analyze the temporal changes in the SDs of the DR values rather than the average DR values, as shown in the analysis in Figure 4. The SDs of the DR values were represented as SD.

Figures S9c and S9d show the temporal changes in the SD at the focal point. Regardless of the laser polarization mode, the SD and the DR decrease (Figures S9a and S9b), indicating an increase in the condensate's anisotropy and a more aligned orientation of the HEWL molecules concerning the laser polarization direction. This observation aligns with the FA results shown in Figure 4. It is crucial to distinguish the symmetry axes governing dipole alignment under distinct polarization modes. For LP light, the symmetry axis corresponds to the vector of linear polarization residing within the plane of the sample. Conversely, for CP light, the symmetry axis aligns with the beam propagation vector, which is orthogonal to the image plane. Furthermore, it is theoretically established that the maximal degree of dipole alignment achievable with CP light is limited to half that attainable with LP light.^[S11,12]^


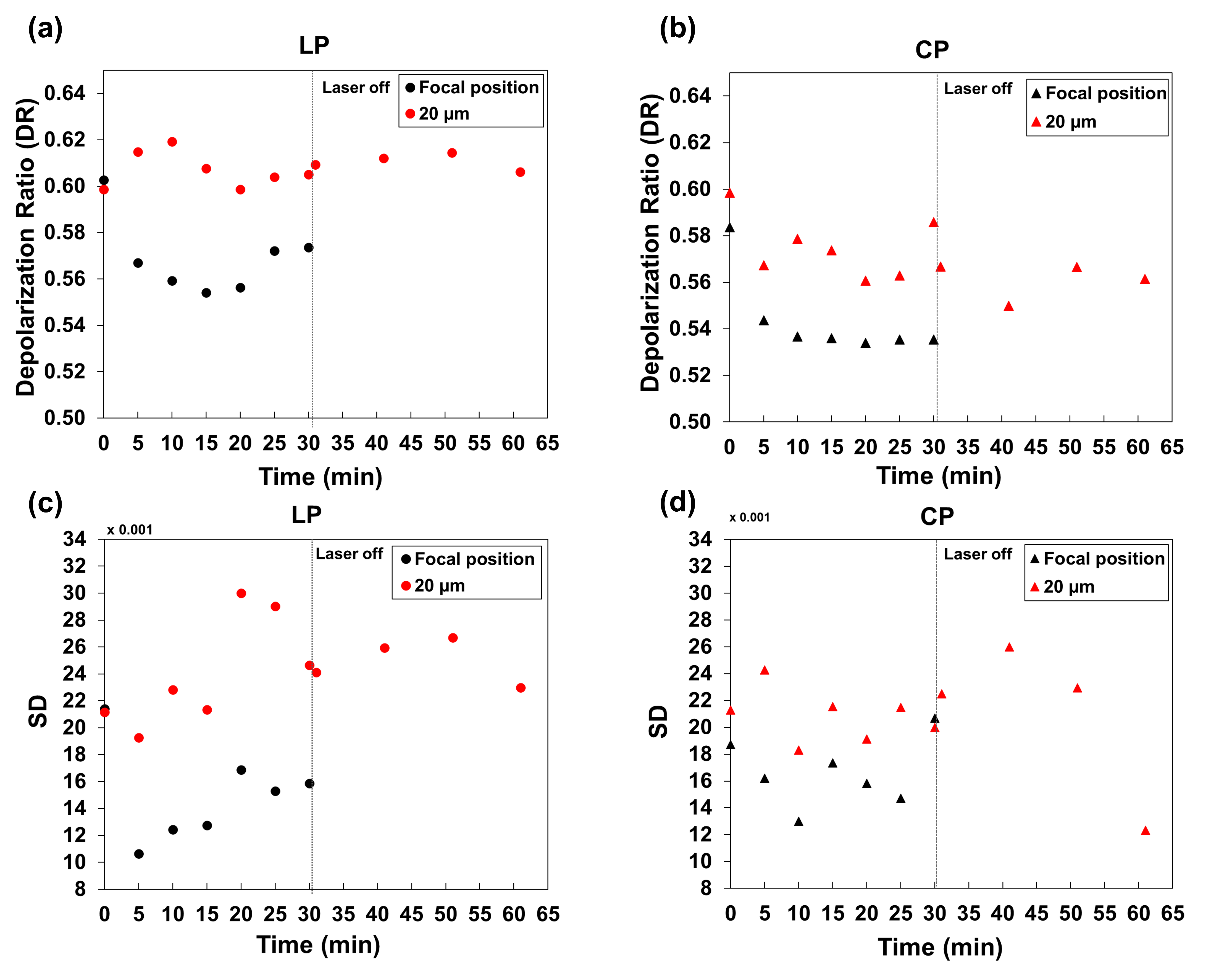


**Figure S9.** Temporal variations in the depolarization ratio (DR) values under (a) LP and (b) CP irradiation conditions. Black circles and triangles denote DR values at the focal position, whereas red circles and triangles indicate DR values at 20 µm away from the focal position. Temporal changes in the SD under (c) LP and (d) CP irradiation conditions. Black circles and triangles represent the SD at the focal position, and red circles and triangles represent the SD at 20 µm away from the focal position.

Next, we analyzed the temporal changes in the SD at 20 μm from the focal point. During the LP trapping laser irradiation, the SD (red circles) increased by approximately 1.5 times over 30 minutes of laser irradiation. Considering the results shown in Figure S9c, an increase in the SD corresponds to greater variation in the DR value, indicating increased anisotropy of the HCD. However, this does not necessarily mean that the orientation of the HEWL molecules aligns with the direction of laser polarization; instead, it could indicate that different orientations in each measurement contribute differently to the DR values. We confirmed that this trend is also observed for well-ordered HEWL crystals when polarized Raman spectroscopy is performed in LP polarization mode without fixing the orientation of the crystals. Notably, the SD remained for 20 min after ceasing laser irradiation, indicating that the anisotropy of the HCD was maintained for approximately 20 min after laser irradiation.

In contrast, during CP trapping laser irradiation, the SD (red triangles) remained almost constant, fluctuating between 21 × 10^−3^ and 26 × 10^−3^, suggesting that the CP laser irradiation has minimal effect on the anisotropy of the HCD. These findings complement the FA measurements presented in Figure 4, reinforcing that the anisotropy of the HCD is attributable to the orientation of the HEWL molecules rather than the fluorescent dye molecules, as determined by polarized Raman measurements. They also suggest that the dye and HEWL molecules are fixed in a specific spatial arrangement.

**References**

[S1] S. Ishizaka, J. Ma, T. Fujiwara, K. Yamauchi, N. Kitamura, *Anal. Sci.* **2016**, *32*, 425.

[S2] K. Setoura, K. Fujita, S. Ito, H. Miyasaka, *J. Nanophotonics* **2018**, *13*, 012504.

[S3] S. Ito, T. Sugiyama, N. Toitani, G. Katayama, H. Miyasaka, *J. Phys. Chem. B* **2007**, *111*, 2365.

[S4] T. Chen, S. Toyouchi, T. Sugiyama, *J. Phys. Chem. C* **2023**, *127*, 23340.

[S5] Ewen Smith, Geoffrey Dent, in *Modern Raman Spectroscopy*, John Wiley & Sons, Ltd, **2019**, pp. 77–99.

[S6] T. Chakraborty, A. L. Verma, *Spectrochim. Acta, Part A* **2002**, *58*, 1013.

[S7] Y. Liu, E. Fratini, P. Baglioni, W.-R. Chen, S.-H. Chen, *Phys. Rev. Lett.* **2005**, *95*, 118102.

[S8] A. Stradner, H. Sedgwick, F. Cardinaux, W. C. K. Poon, S. U. Egelhaaf, P. Schurtenberger, *Nature* **2004**, *432*, 492.

[S9] Y. Harada, T. Asakura, *Opt. Commun.* **1996**, *124*, 529.

[S10] A. Ashkin, J. M. Dziedzic, J. E. Bjorkholm, S. Chu, *Opt. Lett.* **1986**, *11*, 288.

[S11] S. M. Purcell, *Laser Induced Molecular Motion in Strong Nonresonant Laser Fields*, London: University College London, **2010**.

[S12] C. Ellert, P. B. Corkum, *Phys. Rev. A* **1999**, *59*, R3170.
